# Avian Metapneumovirus Subtype B Circulation in Poultry and Wild Birds of Colombia

**DOI:** 10.3390/pathogens13100882

**Published:** 2024-10-10

**Authors:** Santiago Escobar-Alfonso, Diana M. Alvarez-Mira, Magda Beltran-Leon, Gloria Ramirez-Nieto, Arlen P. Gomez

**Affiliations:** 1 Laboratorio de Biología Molecular y Virología, Universidad Nacional de Colombia sede Bogotá, Cra 45 #26-85, Bogotá D.C. 111321, Colombia; dsescobara@unal.edu.co (S.E.-A.); mybeltranl@unal.edu.co (M.B.-L.); gcramirezn@unal.edu.co (G.R.-N.); 2 Laboratorio de Patología Aviar, Universidad Nacional de Colombia sede Bogotá, Cra 45 #26-85, Bogotá D.C. 111321, Colombia; dmalvarezm@unal.edu.co

**Keywords:** *Pneumoviridae*, avian metapneumovirus, avian viruses, avian health, avian pathology, phylogeny

## Abstract

The global poultry industry, as a leading producer of animal protein, faces significant challenges related to animal health and production due to high bird density and disease risks. A major concern is the Avian Respiratory Complex (ARC), a multifactorial health issue involving pathogens such as avian metapneumovirus (aMPV), an often-underdiagnosed component of the ARC. Wild birds are seen as reservoirs and spreaders of the virus. This study aimed to detect the presence and subtypes of aMPV in samples from breeders, broilers, laying hens, and wild birds in Colombia. A total of 273 samples, including swabs from the upper respiratory and reproductive tracts, were collected from commercial poultry and wild birds. Using nested RT-PCR targeting the G gene, aMPV subtype B was identified in 23 samples (8.42%). Sequencing revealed high genetic similarity to vaccine strains, classifying all viruses as vaccine-like. In the commercial birds, aMPV-B appeared in 21 samples, regardless of symptoms, often in tests for other ARC agents, indicating diagnostic bias. In the wild birds, two samples tested positive, suggesting potential transmission between wild and domestic birds. These findings highlight the need for broader diagnostics and further research into aMPV’s impact on avian health.

## 1. Introduction

According to the Food and Agriculture Organization of the United Nations (FAO), the poultry industry is the largest producer of animal protein around the world [1], offering two of the most affordable and protein-rich food sources (eggs and meat) for human nutrition, with a projected growth of 48% in total meat production over the next decade [2]. Therefore, the current state of poultry production worldwide has led to an increase in bird density, accompanied by the corresponding development of new strategies for improving breeding and animal welfare [3]. However, housing a large number of individuals in the same environment is a substantial risk factor for the development of diseases that affect animal health and production, representing a threat to food security.

The Avian Respiratory Complex (ARC) is one of the most significant threats farmers and large-scale producers face in the poultry industry. The ARC is a multifactorial condition in which environmental, management, genetic, and infectious factors converge as causative factors, causing mild to severe respiratory disease in affected birds. Some primary agents may appear without causing an initial alteration or overt disease symptoms, allowing secondary pathogens to enter and produce more extensive damage or alterations that become visible later. Moreover, pathogenicity and clinical manifestations are determined by the interactions among different pathogens within the host [4,5]. The viruses commonly associated with the ARC include Newcastle Disease Virus (NDV), Infectious Bronchitis Virus (IBV), Avian Influenza Virus (AIV), Infectious Laryngotracheitis Virus (ILTV), and avian metapneumovirus (aMPV), while bacteria such as *Mycoplasma synoviae* (MS) and *Mycoplasma gallisepticum* (MG) are often considered primary agents in the ARC [4,6,7].

As a primary agent that has been extensively studied, aMPV is considered the etiological agent responsible for Swollen Head Syndrome (SHS) in chickens and Turkey Rhinotracheitis (TRT) in turkeys. SHS manifestations in chickens are variable, with most cases appearing as imperceptible or asymptomatic disease. When infection is evident, mild respiratory symptoms and reproductive alterations arise, prompting concern. The mild presentation is primarily attributed to a short replication period within the upper respiratory tract tissues of the host. Although severe cases are uncommon, infected birds can develop serious respiratory symptoms, which may also be accompanied by neurological abnormalities [8,9]. Despite this, its significance as a causative or triggering factor in the Avian Respiratory Complex (ARC) is still often underestimated. Secondary infections caused by bacteria such as *E. coli*, *Staphylococcus* spp., and *Pseudomonas* spp. have been reported in birds with the disease [10,11]. Colonization by opportunistic bacteria can exacerbate clinical manifestations, leading to greater tissue damage and reduced viral replication and making virus detection more challenging [12,13]. aMPV is transmitted horizontally and can spread in a flock through contact with secretions, aerosols, contaminated feed, water, and bedding materials. To date, there is insufficient evidence to confirm its vertical transmission [8,13,14]. Furthermore, some studies have confirmed the involvement of wild birds in virus transmission across poultry-farming regions [15,16,17].

aMPV is an enveloped, negative-sense, single-stranded, and unsegmented RNA virus that belongs to the order *Mononegavirales*, the family *Pneumoviridae*, and the genus *Metapneumovirus* [18]. Its genome comprises eight genes encoding eight proteins: nucleocapsid (N), phosphoprotein (P), matrix (M), fusion (F), matrix (M2), small hydrophobic protein (SH), attachment (G), and large polymerase (L). These proteins are organized in the following order: 3′-leader-N-P-M-F-M2-SH-G-L-trailer-5′ [18,19,20]. To date, six subtypes of the virus have been identified: aMPV-A, aMPV-B, aMPV-C, aMPV-D, GuMPV-B25, and PAR-05 [21]. In Latin America, only subtypes A and B have been detected in the commercial and wild birds of Brazil and Mexico, suggesting that these are the most prevalent in the region [22,23,24,25,26].

The importance of aMPV as one of the pathogens of the ARC has often been underestimated, contributing to a diagnostic bias toward other respiratory agents with known prevalence and better-established control strategies when cases of respiratory symptoms are observed in a flock. This bias arises from the absence of concrete evidence linking the virus to clinical signs and insufficient knowledge about the circulating subtypes in poultry and wild birds in specific regions. To address this knowledge gap, the objective of this study was to confirm the presence of aMPV and determine its subtypes in samples obtained from breeders, broilers, laying hens, and wild birds of Colombia through the analysis of the G gene.

## 2. Materials and Methods

### 2.1. Cross-Sectional Study and Samples

A total of 184 samples were selected from clinical cases submitted by the Molecular Biology and Virology Lab (MBVL) of the Universidad Nacional de Colombia (UNAL) for this study based on specific inclusion criteria. These criteria required that the samples were collected in chicken farms, submitted to the MBVL between 2017 and 2023, and sent for molecular diagnostics of various ARC pathogens. Moreover, the samples of interest comprised upper respiratory tract swabs (tracheal, oropharyngeal, or choanal), pooled respiratory organs (trachea and lungs), and reproductive tract imprints (uterus and oviduct), as shown in Table 1. The samples were collected from broilers (*n* = 27), laying hens (*n* = 72), and breeders (*n* = 105) across ten different regions in Colombia known for their high poultry production. Two commercial broiler turkey farms were also included in this study. Ten tracheal swabs were collected from 4-week-old poults on the first farm and another ten tracheal swabs from 14-week-old turkeys on the second. These turkey samples were included in the broiler category. At the time the samples were collected, no live aMPV vaccine was available for use in Colombia.

Furthermore, 69 samples from wild birds of the Wildlife Rescue and Rehabilitation Unit (URRAS) of the UNAL were collected during 2023. Tracheal and oropharyngeal swabs were taken, according to the sizes of the birds, from individuals belonging to 24 species of 11 orders, shown in Table S1.

### 2.2. aMPV Detection Strategy

Viral RNA from the samples was extracted using the High Pure Viral Nucleic Acid Kit^®^ (Roche, Basel, Switzerland), following manufacturer instructions. The RNA extracted from each sample was quantified using the NanoDrop^®^ Lite Plus spectrophotometer (Thermo Fisher Scientific, Waltham, MA, USA). First, complementary DNA (cDNA) synthesis was performed with the OneScript^®^ plus cDNA Synthesis Kit (Applied Biological Materials Inc., Vancouver, BC, Canada). A nested RT-PCR (nRT-PCR) strategy to target the G gene was carried out using the GoTaq^®^ Flexi DNA Polymerase (Promega Corporation, Madison, WI, USA) and two sets of primers that allowed the simultaneous identification of subtypes A and B [27,28]. In the first amplification, the primers G6-(5′-CTGACAAATTGGTCCTGATT-3′) and G1+(5′-GGGACAAGTATCYMKAT-3′) were used. For the nested PCR, forward primers G8+A (5′-CACTCACTGTTAGCGTCATA-3′) and G9+B (5′-TAGTCCTCAAGCAAGTCCTC-3′) were used in combination with the G5-(5′-CAAAGARCCAATAAGCCCA-3′) reverse primer, which anneals with a conserved region of both subtypes. Amplification products were screened using agarose gel electrophoresis. Products with expected sizes of 268 and 366 bp were considered positive for subtypes A and B, respectively. To avoid excluding other subtypes, an RT-PCR assay using primers designed by Chacón et al. [24] to amplify a fragment of the nucleocapsid (N) gene was simultaneously implemented, allowing the detection of all subtypes.

### 2.3. Sequencing and Phylogenetic Analyses

The amplification products of the G gene were purified, and sequences were obtained using the Sanger sequencing platform from these purified products. The obtained sequences were analyzed using the “Nucleotide BLAST”v (2.16.0) tool available from the NCBI (National Center for Biotechnology Information). This preliminary analysis aimed to confirm whether these sequences belonged to the B subtype of the virus and to determine the genetic identity with the established strains published in the NCBI’s GenBank. The G-gene sequences obtained in this study were aligned to 56 aMPV-B strains with the MUSCLE alignment tool available in MEGA 7. European, Brazilian, African, Asian, and North American sequences, including historical B strains (VCO3-60616) and established B vaccine strains (1063 and PL21), were used for phylogenetic analysis.

A phylogenetic tree was constructed using the maximum likelihood method and the Kimura 2-parameter model implemented in MEGA 7. The branch support of the tree was assessed by performing 1000 bootstrap replicates, and only branches with bootstrap values of greater than 70% were considered reliable.

## 3. Results

**Figure 1:**
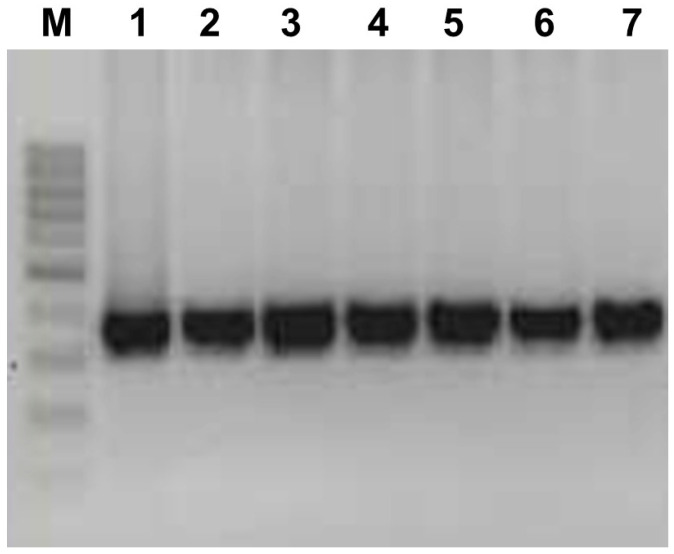
RT-PCR of Colombian aMPV. Lane 1: subtype B vaccine strain. Lanes 2, 3, 4, 5, 6, and 7: detected subtype B strains. M: molecular size marker (100 bp DNA ladder; Thermo Fisher Scientific^®^).

Out of 273 processed samples, aMPV was detected in 23 samples. Each of these positive samples displayed an amplification product of 116 bp on agarose gel electrophoresis for the N gene, as well as a 360 bp product for the G gene, consistent with the positive control of subtype B, as shown in Figure 1. An overall positivity rate of 8.42% was obtained for the evaluated period (2017–2023). Of these samples, 21 (7.69%) belonged to commercial birds while two (0.73%) were from wild birds. No detection of Subtype A or any other subtypes was achieved in any of the samples included in this study.

### 3.1. Detection in Commercial Birds

The virus was detected in 21 samples from three production systems across seven of the ten sampled regions, as detailed in Table 2. Most of the aMPV-positive samples were collected from laying hens, with 11 cases (*n* = 72, 15.28%), eight from breeders (*n* = 105, 7.62%) and two from broiler chickens (*n* = 27, 7.47%). From 21 positive samples, aMPV was detected in 10 originally submitted for the molecular diagnosis of other ARC agents, with only two cases including aMPV in the diagnostic request. The remaining nine samples were solely aimed at diagnosing aMPV.

Furthermore, in three cases, aMPV was detected in samples where other agents were also present. In two of these, MS was previously detected in tracheal swabs from breeders aged 128 and 10 weeks, respectively, through molecular techniques. In the other case, a strain of Infectious Bursal Disease Virus (IBDV) from genogroup 2 was detected in a bursa of Fabricius sample, as requested by the submitter of the clinical case, from the same batch of 35-day-old broiler chickens.

Clinical manifestations associated with aMPV were described in 13 of the 21 positive samples, with the majority of symptoms observed in laying hens, as shown in Table 2. Among these, respiratory rales were the most frequently reported, occurring in six cases either alone or in combination with other respiratory signs, reproductive abnormalities, and one instance of increased mortality. Conversely, unspecified respiratory conditions were reported in five. In three, reproductive abnormalities, including one instance of decreased egg production and two instances of loss of eggshell pigmentation, were reported. The remaining eight (38.10%) did not have clear reports of the signs or irregularities presented by the birds. In six (28.57%), the sample collection was justified as part of a monitoring effort or routine diagnostic for MG-MS in breeders (n = 4, 19.05%), a combination of NDV-IBV-aMPV in breeders (*n* = 1, 4.76%), and IBV in layers (*n* = 1, 4.76%).

The virus was predominantly detected in 17 upper respiratory tract samples (*n* = 175, 9.7%), which accounted for 80.95% of the positive cases. Additional detections were observed in uterine swabs and imprints collected from layer hens (*n* = 29, 10.3%) as well as in a pool of swabs from dead-in-shell (DIS) chicks. The age distribution of samples from long-cycle birds, based on management guides for the most commonly used commercial breeds in Colombia, was as follows: one (4.76%) from nearly hatched chicks, four (19.05%) from birds in the growth stage (7 to 12 weeks), two (9.52%) from birds in the development stage (13 to 18 weeks), five (23.81%) from birds in peak production or phase 1 of production (18 to 40 weeks), four (19.05%) from birds in phase 2 of production (41 to 60 weeks), and three cases (14.29%) from birds in the finishing phase (61 weeks onward). From the short-cycle birds, samples were collected from broiler chicks aged both 35 and 40 days, which are close to the typical age for slaughter. Lastly, in the swabs collected from the DIS chicks, isolation of *Klebsiella* spp. was also reported by a bacteriological culture conducted at another laboratory.

### 3.2. Detection in Wild Birds

From the 69 wild bird samples included in this study, two tested positive for the B subtype of the virus. The first positive result was from a tracheal swab of a stygian owl (*Asio stygius*). The second was a choanal swab obtained from a rock pigeon (*Columba livia*). That bird had an acute respiratory disease being treated at a private clinic in the city. In that case, NDV, ALT, and aMPV detection were requested, with only the latter being detected.

### 3.3. Phylogenetic Analyses

**Figure 2:**
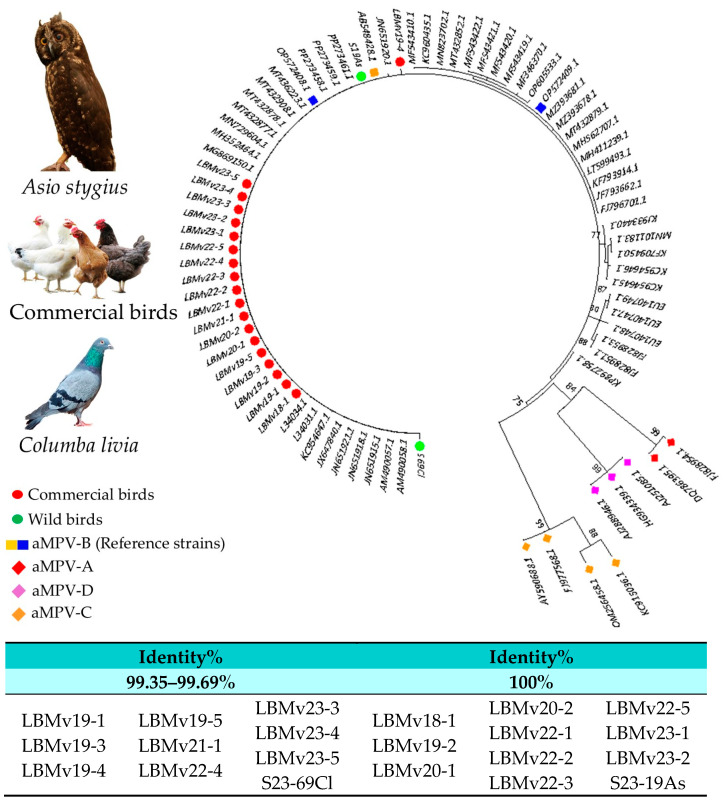
Phylogenetic tree was constructed using partial G-gene sequences of aMPV-B and other subtypes obtained from the NCBI database. Color code circles for the sequences correspond to commercial birds (red), wild birds (green), aMPV-B reference strains (blue and orange squares), and other aMPV subtypes (orange, purple, and red diamonds). Nucleotide sequence identity (NSI) percentages are exhibited by all the obtained strains.

Out of the original 23 positive samples, 21 aMPV-B sequences were obtained and classified following to the criteria proposed by Lupini et al. [29]. Accordingly, viruses were classified as “vaccine-like strains” if the nucleotide sequence identity (NSI) with reference strains 1062 or PL21 exceeded 99.0% and clustered together with either of them. Contrarily, if these viruses did not meet one or both criteria, they were classified as “field strains”. The 21 sequenced viruses were therefore classified as vaccine-like strains, clustering closely with subtype B reference strain 1062 and showing a nucleotide sequence identity (NSI) of over 99.0%, reaching up to 100%, across an average of 325 positions (Figure 2).

## 4. Discussion

aMPV is an underdiagnosed and often underrated agent of the ARC. Its impact on poultry has not been established as clearly as that of other respiratory agents, and its relationship with wild avian species requires further research. Subtype B is considered the most prevalent worldwide, and it spreads primarily throughout the poultry regions of Europe, Asia, Africa, and North and South America. To date, in Central and South America, only Mexico and Brazil have reported the circulation of subtypes A and B in both commercial and wild birds [24,25,26,30]. In Colombia, no studies have been conducted to determine the circulation of any subtypes, their prevalence, or the impact generated by aMPV on the local poultry industry. This study is a first approach to address these knowledge gaps and begin to fill them. Moreover, the recent outbreaks of Highly Pathogenic Avian Influenza (HPAI) in 2022 have been a clear example of the importance and the threat that respiratory diseases pose to the poultry industry, highlighting the not-so-obvious impact on animal welfare and public health [31]. In this sense, the limited understanding of the characteristics of aMPV and the impact it may have on respiratory diseases underscores the need for more comprehensive studies. This is supported by the detection of aMPV-B in samples taken from three poultry production systems, involving wild birds as well, as shown in Table 1 and Table 2, all originating from the regions with the highest poultry densities in the country.

Studies have been conducted in European, African, and Asian countries, where different prevalences of this subtype in commercial birds have been reported). In Europe, Italy has reported a prevalence of 25.6%), while Greece reported 2.8%). In Africa, Ethiopia reported 1.04%, and in Asia, Vietnam reported 17.2% [32,33,34,35]. Brazil was the first country in Latin America to confirm the presence of aMPV-A in broilers and wild birds [36]. Subsequently, individual and dual detection of subtypes A and B were achieved in samples originating from laying hens, broilers, laying turkeys, and breeders [24,25]. In Mexico, only the circulation of aMPV-A in breeders has been reported so far [26]. Contrary to other studies, in the case of Colombia, it is not possible to establish a prevalence that reflects the country’s reality due to the lack of a more extensive probabilistic sampling of the Colombian poultry population. However, confirming the presence and circulation of the virus in broilers, laying hens, breeders, and wild birds is a significant fact that should not be overlooked. It is worth mentioning that contrary to the trend observed in other Latin American countries where aMPV-A appears to be more prevalent, in this study, 100% of the positive samples obtained belonged to aMPV-B.

One interesting finding in this study was the detection of aMPV-B in samples that were originally referred from commercial birds for the diagnosis of other ARC agents, with a few that included aMPV in a list of several other pathogens. These results suggest that there is a diagnostic bias toward ARC agents with known impacts on production, relegating aMPV from the list of possible differential diagnoses when respiratory problems occur on a farm. Some authors emphasize the diagnostic challenges of ARC-related infections due to the varied and nonspecific clinical manifestations that can occur in the field. This highlights the necessity for veterinarians to develop objective and precise detection strategies for each case [3,4]. This could be because infections with subtypes A and B in chickens will frequently not be accompanied by obvious respiratory symptoms, explaining why, in some cases, the virus goes unnoticed on affected farms and its impact is only evident in productive data, such as the growth performance or feed conversion of an infected flock [8]. This phenomenon was also observed in this study, where eight (38.10%) of the positive samples had aMPV detected in birds without any reported symptomatology.

Interestingly, on two occasions, the virus was found in the same sample in which MS was originally detected. This is not unexpected, as a synergism between the two agents when a coinfection of aMPV and MG arises, increasing the invasiveness of the virus and prolonging its replication in a broader range of host tissues, has been described [6,7]. Gobbo et al. 2017 shared the preliminary results of their research on experimental coinfection with MS, confirming that these two agents can prolong the presence of both within the host, comparable to what occurs with MG [37]. On the other hand, the dual detection of aMPV-B and IBDV, as was also found in this study, was previously reported in broilers by Sharifi et al. in 2022 [38]. IBDV infection causes immunosuppression in affected young broilers, which may leave them susceptible to infection with aMPV and the development of respiratory symptoms. These detections and potential coinfections indicate that aMPV may be present even when it is not the primary diagnostic focus. This underscores the importance of incorporating aMPV into comprehensive diagnostic testing in poultry, which should include not only detection techniques but also characterization methods, especially for agents involved in control programs that use live vaccines.

In this study, the virus was detected in swabs from the upper respiratory tract, the pools of respiratory tract organs (tracheas, air sacs, and/or lungs), uterus swabs, reproductive tract imprints, and swabs taken from DIS chicks. As previously described, aMPV detection in upper respiratory tract swabs, as previously described, demonstrates that it remains an effective sample for virus detection. Although virus detections in samples collected from the reproductive tract are not common, they can be beneficial in cases where virus infection is not accompanied by obvious respiratory symptoms or when the virus is no longer in the respiratory system of infected breeders or laying hens. This result is important considering that in the case of Europe, for example, cases have been described where aMPV is attributed to a series of reproductive abnormalities in laying hens without a definitive etiological diagnosis [39]. These findings reflect the diverse clinical presentations of aMPV, ranging from respiratory distress to reproductive issues such as loss of egg pigmentation and decreased egg production. This highlights the virus’s significant impact on affected birds and stresses its potential effects on reproductive health.

The detection of the virus in a sample collected from DIS chicks, in this case, associated with infection by *Klebsiella* spp., was an interesting finding. This is because the possibility of vertical transmission of aMPV has been proposed; however, it has not yet been proven [14]. It is suspected that the virus could have reached the embryo through the contact of the egg with secretions from the potentially infected mother or with contaminated bedding material. Nonetheless, it is not possible to definitively postulate or reject possible vertical transmission because the information is not sufficient to support this theory. On the other hand, while the capacity of aMPV to generate late embryonic death in field conditions is still largely unknown, *Klebsiella pneumoniae* has been recognized as one of the bacterial agents capable of generating this abnormality during embryo development [9,40].

Over the years, multiple studies have been conducted that have proven that chickens of all ages and productive systems are susceptible to aMPV infection [8,9]. Similarly, Lupini et al. detected aMPV-B in vaccinated, and unvaccinated chickens between 15 and 30 days of age, with and without respiratory signs [29]. These results align with the findings of this study, in which the virus was detected in short-cycle birds aged 35 and 40 days, with and without reported respiratory symptoms. In the long-cycle birds, the virus was detected in birds ranging from seven to 128 weeks. It has also been shown that the virus can affect laying hens or breeders from 20 to 70 weeks [39]. The age distribution of the positive samples spanned various stages of bird development, from nearly hatched chicks to birds in the finishing phase of production. This wide range suggests that aMPV can impact birds throughout their life cycles, from the earliest stages of life to the end of their production cycle.

The role played by wild birds in the dissemination of agents causing respiratory diseases has been widely studied since the last century. In the case of aMPV, at the beginning of this century, the ability of populations of wild ducks to become infected and spread, in this case, aMPV-C was evaluated in regions of high turkey production in the United States. Subsequent reports have further confirmed the roles of different orders of wild birds as asymptomatic carriers of the virus [15,30,41]. In Latin America, aMPV-A has been detected in *Anseriformes*, *Columbiformes*, *Falconiformes*, and *Psittaciformes* [22,30]. The present study confirmed the presence of aMPV-B in a pet rock pigeon and a wild stygian owl, which contrasts with the findings of Catelli et al., who stated that pigeons were not susceptible to aMPV-B infection under experimental conditions and therefore do not play an important role in virus dissemination [42]. However, this result agrees with two other studies that also confirmed the opposite. The first study, conducted in Jordan by Gharaibeh and Shamoun in 2012, confirmed the shedding of aMPV-B in young pigeons and house sparrows (Passer domesticus) under experimental conditions. They also reported that none of those birds developed antibodies against the virus 15 days post-infection, concluding that those birds were partially susceptible to the virus and could act as spreaders between poultry farms [43]. The second study was conducted in Brazil by Felippe et al. (2011), where subtypes A and B were detected in samples from feral pigeons that did not show respiratory symptoms at the time of sample collection [30]. Having detected the virus in a pet pigeon raises more questions about the role of that species in the spread of the virus. Furthermore, with just the sample, it was not possible to determine if the virus was the primary agent for the respiratory disease in the bird or if it was involved in a coinfection with other agents. Regarding birds of prey, there is only one report of aMPV-A detection in an American kestrel (*Falco sparverius*) in Brazil. That study presents the first detection of aMPV-B in a nocturnal raptor. That is a nocturnal species that can inhabit urban wooded areas in the city. Its diet is mainly composed of small birds such as pigeons, rails, and cuckoos [44]. With this information, we can propose a hypothesis that an unknown bird came into contact with a nearby poultry farm experiencing an outbreak with the virus, leading to its own infection. This raises the possibility that the pet pigeon or the owl became infected through contact with this unknown bird, either by contact or, in the case of the owl, by consuming an infected bird. The presence of aMPV in these wild birds highlights their potential roles as reservoirs and vectors, which could contribute to the persistence and spread of the virus across different environments and poultry farms. However, respiratory viruses can spread rapidly within a poultry farm and potentially escape to infect local wild birds, thereby expanding their reach and impact beyond the initial outbreak area. With this in mind, in both cases, we propose that the phylogenetic proximity of the analyzed sequences to a vaccine strain may indicate a possible farm origin, highlighting the importance of assessing the presence and impact of shared pathogens between commercial and wild birds. Nonetheless, further testing, such as whole-genome sequencing (WGS), is necessary to validate this hypothesis.

Nguyen et al. [32] analyzed nearly complete sequences of the G gene from circulating aMPV-B strains in Vietnam. This study proposes the existence of a conserved region in approximately the first third of the G-gene sequence (positions 1 to 460) and a more variable region located thereafter (positions 460 to 976). All sequences obtained in this study exhibit a high genetic identity, with the reference subtype B vaccine strain (1062) within the aforementioned conserved region of the G gene, suggesting a certain degree of uniformity among the circulating viruses in Colombia. The primers used in this study successfully amplified a segment of the G gene corresponding to this conserved region [27,28]. While this conserved region facilitates the detection of specific changes and supports confident strain detection methods, its utility for comprehensive comparative studies and phylogenetic analysis should be evaluated. As highlighted by Nguyen et al., incorporating highly divergent regions of the G gene is crucial for accurate genetic characterization and classification. The strategy of amplification of the conserved region of the G gene has been implemented in various studies and provides a preliminary means of differentiating between vaccine-derived and field strains based on minor nucleotide variations relative to a reference strain. However, it may overestimate the reliability of phylogenetic inferences due to the inherent variability of the aMPV G gene. Therefore, while all sequences obtained in this study can be classified as vaccine-like, amplification of the complete G gene or WGS would offer a more comprehensive understanding of the identity of the detected strains and help address the limitations associated with relying solely on conserved regions [29,30,32,45].

However, at the time of this study, no live vaccines have been approved for the control of the virus in the country. Thus, the identification of aMPV-B strains in poultry systems and wild birds will pose several questions about their origin. The scope of this study is not aimed at answering these questions but at presenting relevant information to guide further investigations and hopefully highlight the importance of relevant diseases such as this one.

## 5. Conclusions

The present study confirms the circulation of subtype B of aMPV in commercial and wild birds from regions of poultry production in Colombia. The detection in samples intended for the diagnosis of other agents since 2018 highlights the need to further investigate this virus to better understand its epidemiology and the impact it can generate in the poultry industry and wild birds. The high genetic similarity of the G-gene segments of the detected viruses with a vaccine strain suggests the circulation of vaccine-derived subtype B strains. Therefore, confirming this hypothesis through whole-genome sequencing (WGS) is necessary. The detection in a domestic pigeon and a nocturnal raptor exposed the need to develop additional studies with larger sample sizes to better understand the role of wild birds as possible reservoirs and spreaders of the virus as well as its ecological impact on biodiverse countries. Altogether, this study provides a basis for future research and control actions that will contribute to improving poultry health and the conservation of wild species in these regions.

## Figures and Tables

**Table 1:** Samples from organic systems of commercial (breeders, laying hens, and broilers) and wild birds.

Sampled Organic Systems	Breeders	Laying Hens	Broilers	Wild Birds	Total
Upper respiratory tract	99	49	27	69	244
Reproductive tract	6	23	0	0	29
Total	105	72	27	69	273

**Table 2:** Epidemiological data from the aMPV-B positive samples collected from commercial birds in Colombia.

ID	Year	Age	Sample Type	Initial Diagnostic Objective	Reported Symptoms
**Breeders (*n* = 105, 7.62%)**					
LBMv18-1	2018	Dead-in-shell embryo	Swabs	MG-MS	NR
LBMv19-1	2019	7 weeks	Tracheal swabs	MG-MS	NR
LBMv19-2	2019	36 weeks	Tracheal and air sac swabs	MG-MS-NDV-LTA-IBV-aMPV	Unspecified respiratory symptoms
LBMv19-3	2019	25 weeks	Tracheal swabs	MG-MS	Routine diagnostics
LBMv19-4 ^a^	2019	128 weeks	Tracheal swabs	MG-MS	Routine diagnostics
LBMv19-5	2019	15 weeks	Tracheal swabs	MG-MS	Routine diagnostics
LBMv20-2 ^a^	2020	10 weeks	Tracheal swabs	MG-MS	Routine diagnostics
LBMv22-7	2022	48 weeks	Tracheal swabs	NDV-IBV-aMPV	Routine diagnostics
**Layer hens (*n* = 72, 15.28%)**					
LBMv20-1	2020	33 weeks	Tracheal swabs	NDV-IBV	Increased mortality
LBMv21-1	2021	50 weeks	URT organ pool	IBV	Unspecified respiratory symptoms
LBMv22-1	2022	83 weeks	Uterine swabs	aMPV	Respiratory rales and loss of eggshell pigmentation
LBMv22-2	2022	85 weeks	Uterine swabs	aMPV	Respiratory rales and loss of eggshell pigmentation
LBMv22-3	2022	15 weeks	Tracheal swabs	aMPV	Respiratory rales
LBMv22-4	2022	21 weeks	Tracheal swabs	IBV	Routine diagnostics
LBMv22-5	2022	7 weeks	Infraorbital sinus swabs	aMPV	Respiratory rales, mucus, and facial edema
LBMv22-6	2022	23 weeks	Uterine imprint	aMPV	Respiratory rales and decrease in egg production
LBMv23-1	2023	42 weeks	Tracheal and choanal swabs	IBV	Decrease in egg production
LBMv23-2	2023	25 weeks	Tracheal and choanal swabs	aMPV	Unspecified respiratory symptoms
LBMv23-3	2023	10 weeks	Tracheal swabs	aMPV	Unspecified respiratory symptoms
**Broilers (*n* = 27, 7.41%)**					
LBMv23-4	2023	40 days	Choanal swabs	aMPV	Unspecified respiratory symptoms
LBMv23-5 ^b^	2023	35 days	Choanal swabs	aMPV	Respiratory rales and increased mortality

^a^ Dual detection with *Mycoplasma synoviae*; ^b^ Dual detection with IBDV; NR: no symptoms reported.

## Data Availability

The 21 partial CDS sequences of the aMPV-B attachment glycoprotein gene (19 from chickens and two from wild birds) have been submitted to GenBank and are waiting for accession numbers.

## Supplementary Materials

The following supporting information can be downloaded at: https://www.mdpi.com/article/10.3390/pathogens13100882/s1, Table S1: Orders and species of sampled wild birds.
